# P-68. Comparison of vancomycin vs daptomycin for prosthetic joint infections

**DOI:** 10.1093/ofid/ofae631.275

**Published:** 2025-01-29

**Authors:** Carissa Maria Tedeschi, Douglas Slain, Meera Mehta, Jessica Johnson, Allison Lastinger

**Affiliations:** West Virginia University Hospitals, Westerville, Ohio; University of Pittsburgh, Pittsburgh, Pennsylvania; WVU Medicine, Morgantown, West Virginia; West Virginia University, Morgantown, WV; West Virginia University Hospital, Morgantown, West Virginia

## Abstract

**Background:**

A combination of surgery and extended durations of antimicrobials are the mainstays of therapy for patients with prosthetic joint infections (PJI). In the Infectious Diseases Society of America Clinical Practice Guidelines for Diagnosis and Management of PJI, daptomycin is an ‘alternative regimen’ for patients with methicillin-resistant *Staphylococcus aureus* (MRSA) and other methicillin-resistant coagulase-negative *Staphylococcus* (CoNS) species. The side effect profile of daptomycin may also be more tolerable than that of vancomycin, the preferred agent. This study aims to investigate the tolerability and efficacy of vancomycin versus that of daptomycin for patients with PJI. This topic has not been widely compared in literature for patients with monomicrobial PJIs.

**Methods:**

This was a retrospective, observational assessment of patients who received vancomycin or daptomycin after surgery for prosthetic knee or hip infections. Patients were followed in our University Hospital’s Orthopedic Infectious Diseases Clinic between 1/1/2019 and 4/30/2023. Medical records were reviewed including demographics, type of surgery, adverse effects (AEs), and relapse rates at 6 weeks, 6 months, and one year.

**Results:**

Seventy-nine patients were included: 56 initiated therapy with vancomycin and 23 with daptomycin. The primary outcome was the incidence of therapy-altering AEs. Twenty-two patients in the vancomycin group and 4 patients in the daptomycin group experienced AEs that led to therapy changes (39.3% vs 26.1%, p=0.07). The secondary outcome was the incidence of recurrent infection and was tracked in a subset of 39 vancomycin and 23 daptomycin patients. At 1 year, 10 vancomycin patients required additional unplanned surgery, while only 1 daptomycin patient experienced this (17.9% vs 4.3%, p=0.04). The only statistically significant difference found in baseline characteristics was that more antibiotic spacers were placed in the long-term follow-up daptomycin patients (15 vs 16, p=0.034).

**Conclusion:**

There was no statistically significant difference in tolerating vancomycin vs daptomycin, however, daptomycin may lead to fewer relapses long-term in patients with MRSA and CoNS infections.

**Disclosures:**

**All Authors**: No reported disclosures

